# Additive interaction of snoring and body mass index on the prevalence of metabolic syndrome among Chinese coal mine employees: a cross-sectional study

**DOI:** 10.1186/s12902-019-0352-9

**Published:** 2019-03-04

**Authors:** Yanyan Li, Qian Gao, Lu Li, Yanan Shen, Qing Lu, Jianjun Huang, Chenming Sun, Hui Wang, Nan Qiao, Cong Wang, Haixia Zhang, Tong Wang

**Affiliations:** 1 Department of Health Statistics, School of Public Health, Shanxi Medical University, 56 Xinjiannanlu Street, Taiyuan, Shanxi 030001 People’s Republic of China; 2 Department of Neurosurgery, General Hospital of Datong Coal Mining Group, Datong, China; 3 Department of Urology, General Hospital of Datong Coal Mining Group, Datong, China; 4 Department of Epidemiology and Biostatistics, Michigan State University, East Lansing, MI 48824 USA

**Keywords:** Snoring, BMI, MetS, Additive interaction, Workplace

## Abstract

**Background:**

Although snoring has been previously reported to be associated with metabolic syndrome (MetS), its interaction with body mass index(BMI) on MetS remains unclear. We aimed to examine the individual effects and possible interaction between snoring and BMI on MetS.

**Methods:**

From July 2013 to December 2013, 3794 employees of coal mining enterprises aged 18 to 65 were recruited from Shanxi province of China. The individual effects were assessed by multivariable logistic regression model. Additive interaction was evaluated by calculating the relative excess risk due to interaction (RERI), attributable proportion due to interaction (AP) and synergy index(S).

**Results:**

We found that, after adjusting for potential confounders, odds ratio (OR) and 95% CI for MetS was 1.30 (1.09, 1.56) in occasional snorers and 1.50 (1.24, 1.82) in habitual snorers compared with non-snorers. BMI ≥ 24 was related to high risk of MetS (OR, 3.27; 95% CI, 2.93–3.63). Significant additive interaction between snoring and BMI on MetS was detected. The estimates and 95% CI of the RERI, AP and S were 1.89 (0.67, 3.24), 0.23 (0.08, 0.38), and 1.37 (1.11, 1.75), respectively. However, stratified by workplace, the additive interaction was only significant among underground front-line and ground workers.

**Conclusions:**

Both Snoring and BMI were related to high risk of Mets. Moreover, there are additive interaction between snoring and BMI. Snorers who worked underground front-line and ground are more susceptible to the negative impact of being overweight on MetS.

**Electronic supplementary material:**

The online version of this article (10.1186/s12902-019-0352-9) contains supplementary material, which is available to authorized users.

## Background

Metabolic syndrome, a cluster of metabolic disorders including obesity, high blood pressure, dyslipidemia, and hyperglycemia, is considered to be a risk factor for numerous chronic diseases, such as diabetes, kidney disease, cardiovascular disease, prostatic hyperplasia and subsequent disease-related mortality [1–3]. Given that the prevalence of MetS is increasing in both developed and developing countries and MetS has become a worldwide serious public health problem, identifying simple, feasible and modifiable indicators for screen high-risk group is of critical importance [4, 5]. For high-risk population, we can implement the targeted interventions. It is in conformity with the concept of precision medicine. Coal mine employees, especially the underground front-line workers, exposure to long-term high-risk environment. They are constantly threatened by productive dust (coal dust, silica dust and mixed dust), harmful physical factors (noises, vibration, hot and humid environments), productive poisons and other factors (lead, benzene and trinitrotoluene) [6]. Poor production environment, shift work and frequent coal mine accidents in recent years have led to the occupational stress in coal mine employees. The above factors increase the risk of metabolic syndrome and lead to higher prevalence of metabolic syndrome in coal mine employees than the general population [7]. The potentially useful biomarkers of MetS, play a more valuable role in coal mine employees than in the general population. So we aim to identify indicators of MetS among coal mine employees.

Measuring snoring, a manifestation of obstructive sleep apnea (OSA) syndrome, is easy, low-cost and noninvasive. Cumulative epidemiological data demonstrated that habitual snoring was associated with cardiovascular events [8, 9], hypertension [10], and diabetes [11]. While several studies that focused on the general population have shown that snoring was also strongly related to MetS [12–18]. The association between snoring and MetS is still unknown in coal mine employees. Therefore, in this study we first explored whether the relationship is significant in coal mine employees.

In addition, obesity is a modifiable risk factor of MetS and can be improved through diet control and physical activities. Thus, obesity may be a critical aspect of prevention and management of MetS. Plenty of established evidence have shown that BMI is strongly associated with MetS [19, 20].

Although the exact mechanisms of snoring and BMI on MetS are not fully elucidated, several explanations for the association have been suggested. Hypoxia and hypercapnia induced by snoring stimulate sympathetic nervous activity [21] and increase plasma catecholamine levels [22], which consequently lead to insulin resistance and metabolic disorders [23]. Adipose tissue is a highly active endocrine organ that releases a range of adipokines and promotes expression of inflammatory markers. These inflammatory markers maybe mediate the detrimental effect of obesity on the development of MetS [24].

Based on the above mechanisms, it is plausible to suppose that snoring give rise to the risk of obesity. First, due to the impaired metabolism, snoring may itself predispose individuals to worsening obesity. Furthermore, snoring is the main symptom of OSA. OSA may be associated with changes in hormone level, such as leptin [25], ghrelin [26], and orexin [27]. These hormones can cause increased appetite and calorie intake, thereby promoting obesity. Conversely, obesity also seem to cause and aggravate snoring. Fat deposition in the upper airway lumen and muscle could reduce tracheal traction and lung volume, resulting in the obstruction of upper airway [28]. Thus, it appears that snoring and obesity form a vicious cycle where one results in worsening of the other. In epidemiology, interaction refers to the situation where the effect of one risk factor on a certain disease outcome is different across strata of another risk factor. The joint effect of two factors is not equal to the sum (additive interaction) or the product (multiplication interaction) of individual effect [29]. Therefore, it could be reasonable for us to guess that there is an interaction between snoring and obesity. That is to say, the coexistence of snoring and BMI may synergistically cause MetS.

So far, only one study conducted in a healthy population from China has reported the combined effect of snoring and BMI on MetS [17]. The research found the multiplicative interaction between snoring and BMI in relation to MetS does not exist. However, the absence of multiplicative interaction doesn’t mean there is no additive interaction [29]. Little information about the additive interaction is known. Andersson et al. suggested that we should focus on additive rather than multiplicative interaction when a biologic interaction is examined [30]. Considering additive interaction is more closely relevant to prevention and risk prediction of diseases, we decided to examine whether the additive interaction between snoring and BMI exist.

Furthermore, long-term occupational exposure, such as coal dust, may affect the worker’s airway conditions and lipid metabolism [31]. And workers in different workplace were exposed to different occupational risk factors. Therefore, this study also aimed to explore whether the result of additive interaction is consistent among coal mine employees in different workplace.

## Methods

### Study population

The study subjects were recruited from a large coalmine population with more than 200,000 samples in Shanxi Province of China, from July 2013 to December 2013. This survey was conducted with a two-stage cluster stratified population sampling method. In the first stage, ten coalmines were randomly selected from 87 coalmines. We got the list of the 87 coalmines from general Hospital of Datong Coal Mining Group. All of the 10 coalmines that randomly drew from the list agreed to join our study and we got the informed consent from management of the coalmines. In the second stage of sampling, employees of coal mining enterprises were selected by the stratified random sampling method based on the baseline data including date of birth, gender, and work type, which were provided by the management of the coalmine group.

The sample size was computed according to the following formula:

1 $$ n={U}^2\pi \left(1-\pi \right)/{\delta}^2 $$

2 $$ {n}_c=\frac{n}{1+n/N} $$

3 $$ {n}_c=3755 $$

Where, *U* is the two-tailed standard normal variate value related to the null hypothesis, and *π* is prevalence of MetS, *δ* is allowable error. *N* is the population of the coal mine. Considering the expected prevalence of MetS of 33.9% [32], type I error of 0.05, allowable error of 0.015, 3755 subjects would be necessary for the study. Taking no response from some subjects or other non-conforming situations into consideration, we aimed to survey 4600 coal mine employees. Among these individuals, 4298 subjects agreed and completed the study, for a response rate of 93.43%. Besides, five hundred and four subjects who had missing values for variables of interest were excluded from the main analysis, and missing data were filled with multiple imputation (MI) method to conduct the sensitivity analysis.

### Assessment of snoring and related factors

This survey was conducted by face-to-face interviews and by well-trained interviewers with medical knowledge. The questionnaires in our study consisted of questions on demographics, lifestyle and medical history factors. Snoring status was obtained from the question “Have you ever snored during sleep?” with three choices (“never”, “occasionally”, “habitually”). “Occasionally” refers to 1–2 days per week; “habitually” refers to ≥3 days per week. This question is answered by the participants themselves or with the assistance of their families. The following variables were assessed by three categories: family income (≤4000, 4000–6000, ≥6000CNY/month), educational level (bachelor degree or above, junior college and senior high school, and junior high school or below), marital status (single, married and divorced), work type (heavy physical, light physical, and mental labor). Current smoking and alcohol consumption were defined as binary variables (yes, no). The international physical activity questionnaire (IPAQ) was used to assess the physical activity level. Physical activity level was grouped as inactive, minimally active and health-enhancing physical activity [33]. Workplace was grouped as underground front-line, underground auxiliary, ground and office. Underground front-line work includes machinery driving, manuals driving, artillery mining and reserves. Underground auxiliary mainly includes preliminary safety checking, ventilation, and recovery maintenance. Ground work includes transportation, communication, operation management and power supply. Office work includes human resources, administration and other supporting departments.

### Anthropometrics variables and biochemical variables

The anthropometrics variables including height, body weight and waist circumference were measured multiple times by trained and certified examiners to ensure accuracy. Participants were lightly dressed without shoes when height and body weight were measured. The measuring accuracy for height and weight were 0.1 cm, 0.1 kg, respectively. BMI was calculated as weight (kg) divided by height squared (m^2^) and was classified to 2 categories (BMI < 24; BMI ≥ 24). Waist circumference (WC) was measured to the nearest 0.1 cm at the midpoint between the lowest rib margin and the iliac crest during expiration [34]. Participants’ blood pressure was measured on the right upper arm, using a standard mercury sphygmomanometer by the nursing staff. Both systolic blood pressure (SBP) and diastolic blood pressure (DBP) were measured 3 times at 2-min intervals with study subjects in the sitting position after resting for 5 min.

Blood samples taken from the antecubital vein were collected in the morning after a 10-h overnight fast. Concentrations of TG, HDL-C and fasting plasma glucose were measured by the SIEMENS ADVIA 1800 Automatic Biochemical analyzer (JEOL Ltd., Tokyo, Japan).

### Definition of metabolic syndrome

MetS was defined by the harmonized criteria recommended by the 2009 International Diabetes Federation (IDF). According to the criteria, reaching any three of the following five factors will be diagnosed as MetS: (1) elevated TG: ≥1.7 mmol/L; (2) high blood pressure: ≥130/85 mmHg; (3) T_2_DM or fasting plasma glucose: ≥5.6 mmol/L; (4) reduced HDL-C: < 1.0 mmol/L(men) and < 1.3 mmol/L (women); and (5) elevated waist circumference(WC):WC ≥ 85 cm and 80 cm in Chinese men and women, respectively [35].

### Statistical analysis

All data were double-entered into Epi info version 3.5.1 (CDC, Atlanta, USA) to reduce potential errors when we prepared the dataset for statistical analysis. All general characteristics were categorical data, which were described by frequencies and percentages. The univariate logistic regression was used to examine the relationship between independent variables and MetS. Crude odds ratios (cORs) and 95% confidence intervals (95% CI) were calculated. The effects of snoring status and BMI on metabolic syndrome were analyzed, using two different multiple logistic regression models. The first model was adjusted for age and sex. Besides age and sex, the second model was also adjusted for current smoking, alcohol consumption, marital status, physical activity level, educational level, monthly income, and work type. When exploring the relationship between snoring and Mets components, the univariate and multiple logistic regression models were used. The aforementioned analyses were performed using PROC LOGISTIC in SAS 9.2 software (SAS Institute, Inc., Cary, NC, USA). Variance inflation factor (VIF) was used to assess potential collinearity among variables.

The additive interaction between snoring(no vs. yes) and BMI(< 24 vs. ≥24 kg/m^2^) was evaluated by three measures: relative excess risk due to interaction (RERI), attributable proportion (AP) and synergy index (S) [30, 36]. In this study, we took the “both non-snorers and BMI<24” as the reference group, OR_11_ refers to the effect of Mets for snorers whose BMI ≥ 24; OR_10_ represents the effect of Mets for non-snorers whose BMI ≥ 24; while OR_01_ is the effect for snorers whose BMI < 24. Then, RERI = OR_11_-OR_10_- OR_01_ + 1; AP = RERI/OR_11_; S = (OR_11_–1)/[(OR_01_–1) + (OR_10_–1)]. We adopted the bootstrap percentile method which was presented by Knol et al. to calculate the corresponding 95% CI for the estimates of interaction [19]. If there was no additive interaction, the 95% CI of RERI and AP would include 0, whereas 95% CI of S would contain 1. R 3.2.3 was used to carry out the bootstrap procedure. In this analysis, all tests were 2-sided, and a *P*-value of < 0.05 was considered statistically significant.

## Result

### Characteristics of the study population

The prevalence of MetS in this study was 38.88%. The proportion of occasional and habitual snoring in the study were 37.35 and 27.39%, respectively. Over half (58.51%) of individuals were either overweight or obese. According to the results of univariate logistic regression analysis, all independent variables except educational level were related to the prevalence of MetS (see Table 1).

**Table 1:** Characteristics of the study population and their association with MetS

Characteristics	Mets		OR	*P* value
	Yes (1475)	No (2319)		
Gender, *n* (%)				
Men	1306 (88.54)	1891 (81.54)	1.75 (1.44,2.12)	< 0.001*
Women	169 (11.46)	428 (18.46)		
Age			1.42 (1.31,1.55)	< 0.001*
≤ 35	296 (20.07)	679 (29.28)		
35–45	502 (34.03)	855 (36.87)		
≥ 45	677 (45.90)	785 (33.85)		
Monthly income (CNY), *n* (%)			0.91 (0.84,0.99)	0.039*
≤ 4000	416 (28.20)	573 (24.71)		
4000–6000	625 (42.37)	1025 (44.20)		
≥ 6000	434 (29.42)	721 (31.09)		
Marital status, *n* (%)				
Single	36 (2.44)	142 (6.12)	1 (reference)	
Married	1415 (95.93)	2136 (92.11)	2.61 (1.80,3.79)	< 0.001*
Divorced	24 (1.63)	41 (1.77)	2.31 (1.24,4.30)	0.008*
Educational level, n(%)				
Junior high school or below	420 (28.47)	627 (27.04)	1 (reference)	
Bachelor degree or above	172 (11.66)	280 (12.07)	0.92 (0.73,1.15)	0.453
Junior college and senior high school	883 (59.86)	1412 (60.89)	0.93 (0.80,1.08)	0.366
Work type, *n* (%)				
Heavy physical	316 (21.42)	654 (28.20)	1 (reference)	
Light physical	795 (53.90)	1076 (46.40)	1.53 (1.30,1.80)	< 0.001*
Mental labor	364 (24.68)	589 (25.40)	1.28 (1.06,1.54)	0.010*
Current smoking, *n* (%)				
Yes	875 (59.32)	1293 (55.76)	1.16 (1.01,1.32)	0.031*
No	600 (40.68)	1026 (44.24)		
Alcohol consumption, *n* (%)				
Yes	676 (45.83)	886 (38.21)	1.37 (1.20,1.56)	< 0.001*
No	799 (54.17)	1433 (61.79)		
Physical activity level, *n* (%)				
Inactive	134 (9.08)	161 (6.94)	1 (reference)	
Minimally Active	452 (30.64)	657 (28.33)	0.83 (0.64,1.07)	0.149
Health-enhancing physical activity	889 (60.27)	1501 (64.73)	0.71 (0.56,0.91)	0.006*
Workplace				
Underground front-line	237 (16.07)	525 (22.64)	1 (reference)	
Underground auxiliary	538 (36.47)	792 (34.15)	1.51 (1.25,1.82)	< 0.001*
Ground worker	421 (28.54)	561 (24.19)	1.66 (1.36,2.03)	< 0.001*
Office worker	279 (18.91)	441 (19.01)	1.56 (1.21,2.00)	0.139
Snoring, *n* (%)				
Never	375 (25.42)	960 (41.40)	1 (reference)	
Occasionally	556 (37.69)	864 (37.26)	1.65 (1.40,1.93)	< 0.001*
Habitually	544 (36.88)	495 (21.35)	2.81 (2.37,3.34)	< 0.001*
BMI (kg/ m2), *n* (%)				
< 24	1305 (56.27)	269 (18.24)		
≥ 24	1014 (43.73)	1206 (81.76)	5.77 (4.94,6.74)	< 0.001*

*MetS* metabolic syndrome

*is used to highlight statistically significant (*p* < 0.05) findings

### Association between snoring and MetS

The association between snoring and Mets is shown in Table 2. There was no evidence of multi-collinearity as all VIF values were less than 10. For both multiple logistic regression models with different covariates adjustment, we found snoring is strongly associated with MetS and the risk of MetS increased progressively according to snoring status. In model 1, the ORs for MetS were 1.64 (1.39, 1.92) in occasional snorers and 2.50 (2.10, 2.97) in habitual snorers compared with non-snorers. However, with additional adjustment for current smoking, alcohol consumption, marital status, physical activity level, monthly income, work type, and BMI, the ORs of MetS for snorers were attenuated but remained significant for occasional snoring (OR, 1.31; 95% CI, 1.09–1.56) and habitual snoring (OR, 1.50; 95% CI, 1.24–1.82). We also explored the association between snoring and MetS using two multiple logistic regression models, which included snoring as binary variable. The OR of MetS for individuals with snoring was 1.38 (1.17, 1.62), compared with non-snorers**.**

**Table 2:** Odds Ratios and 95% confidence intervals for the presence of MetS according to snoring status and BMI

	Snoring status			Snoring		BMI	
	Never	Occasionally	Habitually	No	Yes	<24	≥24
MetS, *n* (%)	375 (28.09)	556 (39.15)	544 (52.36)	375 (28.09)	1100 (44.73)	1574 (17.09)	2220 (54.32)
Model 1	1.00	1.64 (1.39,1.92) *P* < 0.001	2.50 (2.10,2.97) *P* < 0.001	1.00	1.95 (1.69,2.26) *P* < 0.001	1.00	5.49 (4.70,6.43) *P* < 0.001
Model 2	1.00	1.31 (1.09,1.56) *P* = 0.0034	1.50 (1.24,1.82) *P* < 0.001	1.00	1.38 (1.17,1.62) *P* < 0.001	1.00	5.18 (4.41,6.08) *P* < 0.001

Model 1: adjusted for age and sex

Model 2: adjusted for age, sex, current smoking, alcohol consumption, marital status, physical activity level, monthly income, work type. Additionally, BMI was included as a covariate when assessing snoring status and snoring status as a covariate when assessing BMI

### Association between snoring and MetS components

The associations between snoring and MetS components are shown in Table 3. The prevalence of MetS components increased gradually according to snoring status in both univariate and multiple logistic regression models. After further adjusted for additional confounding factors, the association between snoring and high blood pressure, elevated fasting glucose, hypertriglyceridemia, abdominal obesity were attenuated but remained significant. However, the association with low HDL was no longer significant.

**Table 3:** Odds Ratios and 95% confidence intervals for the presence of metabolic syndrome components according to snoring status and BMI

	Snoring status			BMI	
	Never	Occasionally	Habitually	< 24	≥24
High blood pressure					
Model 1	1.00	1.18 (1.02,1.37)	2.04 (1.73,2.40)	1.00	2.03 (1.78, 2.32)
Model 2	1.00	1.16 (0.99,1.35)	1.70 (1.43,2.02)	1.00	1.85 (1.62, 2.12)
Model 3	1.00	1.02 (0.87,1.19)	1.30 (1.09,1.56)	1.00	1.73 (1.51, 1.99)
Elevated fasting glucose					
Model 1	1.00	1.21 (0.97,1.52)	2.15 (1.73,2.68)	1.00	2.16 (1.78, 2.63)
Model 2	1.00	1.22 (0.97,1.53)	1.74 (1.39,2.18)	1.00	1.94 (1.59, 2.36)
Model 3	1.00	1.09 (0.85,1.37)	1.39 (1.09,1.79)	1.00	1.78 (1.45, 2.19)
Hypertriglyceridemia					
Model 1	1.00	1.39 (1.19,1.63)	2.39 (2.02,2.82)	1.00	3.83 (3.32, 4.43)
Model 2	1.00	1.33 (1.13,1.55)	2.07 (1.75,2.46)	1.00	3.59 (3.11,4.16)
Model 3	1.00	1.07 (0.91,1.27)	1.36 (1.13,1.64)	1.00	3.35 (2.88.3.90)
Low HDL-cholesterol					
Model 1	1.00	1.15 (0.97,1.35)	1.33 (1.12,1.59)	1.00	2.37 (2.04, 2.76)
Model 2	1.00	1.23 (1.04,1.45)	1.58 (1.32,1.90)	1.00	2.77 (2.36, 3.24)
Model 3	1.00	1.08 (0.90,1.29)	1.16 (0.95,1.42)	1.00	2.71 (2.31,3.20)
Abdominal obesity					
Model 1	1.00	1.98 (1.67,2.35)	4.24 (3.38,5.31)	1.00	15.55 (12.61, 19.17)
Model 2	1.00	1.94 (1.63,2.31)	3.74 (2.97,4.71)	1.00	14.85 (12.03, 18.33)
Model 3	1.00	1.52 (1.25,1.83)	1.97 (1.51,2.56)	1.00	13.65 (11.01, 16.92)

Model 1: not adjusted for other covariates

Model 2: adjusted for age and sex

Model 3: adjusted for age, sex, current smoking, alcohol consumption, marital status, physical activity level, monthly income, work type. Additionally, BMI was included as a covariate when assessing snoring status and snoring status as a covariate when assessing BMI

### Association between BMI and MetS/MetS components

Our study indicated that BMI was strongly related to MetS. As shown in Table 1, the crude OR between BMI and MetS was 5.77 (4.94, 6.74). If additional covariates, significant in table1, were further adjusted, the OR for BMI reduced to 5.18 (4.41, 6.08). BMI’s association with high blood pressure, elevated fasting glucose, hypertriglyceridemia, low HDL and abdominal obesity were significant (see Table 3).

### The additive interaction between snoring and BMI

Participants were divided into 4 subgroups according to snoring and BMI level (Table 4). Compared with non-snorers with BMI < 24, the multi-adjusted ORs were 5.57(4.24, 7.31), 1.62(1.23, 2.14), and 8.09(6.36, 10.29) for non-snorers with BMI ≥ 24, snorers with BMI < 24, and snorers with BMI ≥ 24, respectively. There was a significant additive interaction between snoring and BMI on MetS in this multi-adjusted model (Table 4). RERI was 1.89, suggesting that there would be 1.89 relative excess risk due to the additive interaction. We also found that 23% of the OR of being MetS exposed to both risk factors was attributable to the additive interaction, and the risk of MetS in snorers with BMI ≥ 24 was 1.37 times as high as the sum of risks in the participants exposed to a single risk factor alone. More specifically, snoring and BMI acted synergistically in the prevalence of MetS. In other words, these two factors interact to increase the risk of MetS, an effect that is more than summation of individual effects.

**Table 4:** Additive interactions between snoring and BMI

		Interaction Analysis				
Snoring	BMI	MetS, *n* (%)	Multi-adjusted OR (95% CI)	RERI (95% CI)	AP (95% CI)	S (95% CI)
All workers						
No	<24	96 (13.10)	1.00	1.89* (0.67, 3.24)	0.23* (0.08, 0.38)	1.37* (1.11, 1.75)
	≥24	279 (46.35)	5.57 (4.24, 7.31)			
Yes	<24	173 (20.57)	1.62 (1.23, 2.14)			
	≥24	927 (62.85)	8.09 (6.36, 10.29)			
Underground front-line						
No	<24	9 (6.16)	1	6.67* (1.42, 19.42)	0.41* (0.10, 0.62)	1.76* (1.11, 2.98)
	≥24	47 (34.81)	8.23 (3.81, 17.80)			
Yes	<24	27 (14.36)	2.52 (1.34, 5.56)			
	≥24	154 (52.56)	16.43 (8.00, 33.73)			
Underground auxiliary						
No	<24	31 (13.08)	1	0.32 (−3.90, 3.25)	0.04 (−0.34, 0.32)	1.04 (0.72, 1.58)
	≥24	89 (50.57)	7.36 (4.51, 12.03)			
Yes	<24	68 (22.97)	1.97 (1.23, 3.15)			
	≥24	350 (56.36)	8.65 (5.71, 13.10)			
Ground workers						
No	<24	37 (16.82)	1	2.52* (0.08, 5.35)	0.32* (0.01, 0.56)	1.59* (1.02, 2.72)
	≥24	80 (52.95)	4.78 (2.95, 7.75)			
Yes	<24	47 (23.62)	1.47 (0.90, 2.41)			
	≥24	255 (62.05)	7.77 (5.10, 11.86)			
Office workers						
No	<24	19 (14.62)	1	1.10 (−1.22, 3.31)	0.21 (−0.24, 0.50)	1.35 (0.77, 2.76)
	≥24	63 (45.99)	3.92 (2.13, 7.23)			
Yes	<24	31 (19.75)	1.18 (0.62, 2.25)			
	≥24	166 (56.08)	5.19 (2.94, 9.17)			

*is used to highlight statistically significant (p < 0.05) findings

We further analyzed the additive interaction across different workplace, and found the inconsistent results. The additive interaction effect was significant among underground front-line workers (RERI: 6.67 (1.42, 19.42); AP: 0.41 (0.10, 0.62); S: 1.76 (1.11, 2.98)) and ground workers (RERI: 2.52 (0.08, 5.35); AP: 0.32 (0.01, 0.56); S: 1.59 (1.02, 2.72)).While it was not significant among underground auxiliary workers and office workers.

### The result of sensitivity analysis

As shown in the appendix table1, the demographic characteristics of the “before MI” and “after MI” group were not statistically significant (Additional file 1: Table S1). And the additive interaction is consistent in the two groups (Additional file 2: Table S2).

## Discussion

Our study explored the individual effect and interactive effect between snoring and BMI on MetS. We found that both Snoring and BMI were related to Mets. We also suggest an additive interaction between snoring and BMI on MetS among employees of Datong coal mining enterprises. From the result of subgroup analysis, the additive interaction effect remained significant among underground front-line workers and ground worker.

The relationship between snoring and MetS has been explored in previous studies. Leineweber et al. reported that snoring frequency was independently associated with MetS in Swedish middle-aged women, after adjustment for potential confounding factors [13]. A cross-sectional study conducted in India also showed that snoring was significantly associated with MetS even after adjusting for age, sex, family history of diabetes, physical activities, smoking, and alcohol [14]. Sabanayagam et al. observed a positive association between snoring and MetS in a representative sample of US adults [15]. Our data suggested that snoring is significantly associated with MetS, and the association is generally in line with current evidence from other population groups in both developed and developing countries [12–18].

Furthermore, we also observed strongly positive associations between snoring status and MetS components including high blood pressure, elevated fasting glucose, hyper-triglyceridemia, and abdominal obesity. However, a Korean multi-rural communities cohort study indicated that snoring frequency was associated with high blood pressure, elevated glucose, and abdominal obesity, but not associated with hypertriglyceridemia and low HDL cholesterol [16]. A prospective study conducted in the United States revealed that loud snoring was a significant predictor of hyperglycemia and low HDL [18]. The discrepancies among studies might be due to differences in the measurement of snoring status, ethnics, and study design.

In current study, we also found that BMI was associated with a higher risk of MetS. Many previous studies have demonstrated this association [17, 18]. A research conducted among Mexico undergraduate students concluded that BMI was a valuable indicator to estimate MetS prevalence [19]. Yang et al. found that BMI showed a dose-response association with the increased risk of MetS [20].

To the best of our knowledge, this study is the first to examine the possible additive interaction between snoring and BMI on MetS. Results of our study revealed that the effect of snoring on MetS was significantly modified by obesity, which was consistent with an additive interaction [36]. The RERI of 1.89 means that the relative risk of having MetS in snores with BMI ≥ 24 is 1.89 higher than the sum of the independent ORs for being overweight alone and snoring alone. That is to say, the risk conferred by one of the factors is increased by the presence of the other. Therefore, snorers should pay more attention to controlling weight in order to prevent MetS and subsequent diseases.

Further stratified by workplace, we obtained more accurate information. The interaction effect was significant among underground front-line and ground workers. While it was not significant among underground auxiliary, and office workers. This may be related to some sort of occupational exposure, such as coal dust. Certainly, further studies and measurement of biomarkers of exposure are needed to determine what caused the difference.

It is beneficial to use snoring and BMI as the easily measured, low-cost and non-invasive indicators during the screening of individuals who are prone to MetS, particularly among underground front-line and ground workers. A doctor of respiratory medicine is suggested to observe the patient’s BMI and metabolic syndrome-related indicators meanwhile in the diagnosis of sleep apnea syndrome, so that we can achieve early detection of patients or high-risk individuals with MetS. Because snorers with overweight or obesity have the highest risk for MetS, underground front-line workers and ground workers in this subgroup may benefit most from a targeted intervention or treatment.

Limitations of this study should also be considered. Firstly, the cross-sectional design did not allow us to infer causality. Secondly, the underreporting of snoring might lead to misclassification bias and weaken the association between snoring and MetS. Thirdly, as with any observational studies, we could not exclude the possible effects of uncontrolled or inadequately measured confounders on the results. Despite of these limitations, this is the first study, to our knowledge, to examine the additive interaction between snoring and BMI on MetS. Further prospective cohort studies with large sample sizes are necessary to reconfirm this additive interaction and to elucidate the underlying mechanism that will eventually contribute to more effective prevention strategies on MetS.

## Conclusion

In conclusion, results from this cross-sectional study among Chinese coal miners confirmed previous findings on association of snoring and BMI with MetS. Moreover, this study further demonstrated an additive interaction between snoring and BMI on MetS in underground front-line and ground workers, highlighting that snorers who worked underground front-line and ground are more susceptible to the negative impact of being overweight on MetS. Preventive strategies aimed at reducing the prevalence of MetS among underground front-line and ground workers are necessary for target snorers with overweight.

## Additional files

Additional file 1: **Table S1.** Characteristics of the study population (before and after multiple imputation). **Table S2.** Additive interactions evaluated by multiple imputated data set. (DOCX 28 kb)

Additional file 2: Variable Assignment Table. (DOCX 17 kb)

## Abbreviations

AP: Attributable proportion due to interaction
BMI: Body mass index
DBP: Diastolic blood pressure
HDL-c: High-density lipoprotein cholesterol
IDF: The International Diabetes Federation
MetS: Metabolic Syndrome
MI: Multiple imputation
OSA: obstructive sleep apnea
RERI: Relative excess risk due to interaction
S: Synergy index
SBP: Systolic blood pressure
T2DM: Type 2 diabetes mellitus
TC: Total cholesterol
TG: Triglycerides
WC: Waist circumference
