# Supplementary material for: Additive interaction of snoring and body mass index on the prevalence of metabolic syndrome among Chinese coal mine employees: a cross-sectional study
Source: BMC Endocr Disord. 2019 Mar 4;19:28. doi: 10.1186/s12902-019-0352-9 (PMC6399959; doi:10.1186/s12902-019-0352-9)
Supplement: Supplementary file 1 — Table S1. Characteristics of the study population (before and after multiple imputation). Table S2. Additive interactions evaluated by multiple imputated data set. (DOCX 28 kb) [file 12902_2019_352_MOESM1_ESM.docx]

**Table S1: Characteristics of the study population (before and after multiple imputation)**

| Characteristics | Before MI  (n=3794) | After MI  (n=4298) | $\chi^{2}$ | P  value |
| --- | --- | --- | --- | --- |
| MetS, n(%) |  |  | 0.53 | 0.46 |
|  | 1475(38.88) | 1637(38.09) |  |  |
|  | 2319(61.12) | 2661(61.91) |  |  |
| Gender, n(%) |  |  | 0.01 | 0.99 |
| Men | 3197(84.26) | 3622(84.27) |  |  |
| Women | 597(15.74) | 676(15.75) |  |  |
| Age |  |  | 0.30 | 0.86 |
| ≤35 | 962(25.36) | 1067(24.83) |  |  |
| 35-45 | 1370(36.11) | 1563(36.37) |  |  |
| ≥45 | 1462(38.53) | 1668(38.81) |  |  |
| Monthly income(CNY),n(%) |  |  | 3.24 | 0.19 |
| ≤4000 | 989(26.07) | 1183(26.36) |  |  |
| 4000-6000 | 1650(43.49) | 1806(42.02) |  |  |
| ≥6000 | 1155(30.44) | 1359(31.62) |  |  |
| Marital status,n(%) |  |  | 0.11 | 0.95 |
| Single | 178(4.69) | 195(4.54) |  |  |
| Married | 3551(95.31) | 4029(93.74) |  |  |
| Divorced | 65(1.71) | 74(1.72) |  |  |
| Educational level,n(%) |  |  |  |  |
| Junior high school or below | 1047(27.60) | 1200(27.92) | 0.11 | 0.95 |
| Bachelor degree or above | 452(11.91) | 509(11.84) |  |  |
| Junior college and senior high school | 2295(60.49) | 2589(60.24) |  |  |
| Work type,n(%) |  |  |  |  |
| Heavy physical | 970(25.57) | 1126(26.20) | 0.42 | 0.81 |
| Light physical | 1871(49.31) | 2101(48.88) |  |  |
| Mental labor | 953(25.12) | 1071(24.92) |  |  |
| Current smoking,n(%) |  |  |  |  |
| Yes | 2168(57.14) | 2476(57.61) | 0.18 | 0.67 |
| No | 1626(42.86) | 1822(42.39) |  |  |
| Alcohol consumption,n(%) |  |  |  |  |
| Yes | 2232(58.83) | 2516(58.54) | 0.07 | 0.79 |
| No | 1562(41.17) | 1782(41.46) |  |  |
| Physical activity level,n(%) |  |  |  |  |
| Inactive | 295(7.78) | 340(7.91) | 0.06 | 0.97 |
| Minimally Active | 1109(29.23) | 1258(29.27) |  |  |
| Health-enhancing physical activity | 2390(62.99) | 2700(62.82) |  |  |
| Workplace |  |  |  |  |
| Underground front-line | 762(20.08) | 901(20.96) | 6.47 | 0.09 |
| Underground auxiliary | 1331(35.08) | 1475(34.32) |  |  |
| Ground worker | 981(25.86) | 1115(25.94) |  |  |
| Office worker | 620(18.98) | 807 (18.77) |  |  |
| Snoring, n (%) |  |  |  |  |
| No | 1335(35.19) | 1518(35.32) | 0.02 | 0.90 |
| Yes | 2459(64.81) | 2780(64.68) |  |  |
| BMI(kg/ m2) , n (%) |  |  |  |  |
| <24 | 1574(41.49) | 1802(41.93) | 0.16 | 0.69 |
| ≥24 | 2220(58.51) | 2496(58.07) |  |  |

The demographic characteristics of the two groups were not statistically significant.

**Table S2: Additive interactions evaluated by multiple imputated data set**

|  |  |  |  | | Interaction Analysis | | | |
| --- | --- | --- | --- | --- | --- | --- | --- | --- |
|  | Snoring | BMI | MetS,n (%) | | Multi-adjusted OR (95% CI) | RERI  (95% CI) | AP  (95% CI) | S  (95% CI) |
| All Workers | | | | | | | | |
|  | No | <24 | 114(13.56) | | 1.00 | **1.84**  **(0.72,2.97)** | **0.25**  **(0.10,0.37)** | **1.40**  **(1.13,1.74)** |
|  |  | ≥24 | 307(45.35) | | 5.14(3.99,6.61) |  |  |  |
|  | Yes | <24 | 193(20.08) | | 1.51(1.17,1.95) |  |  |  |
|  |  | ≥24 | 1023(56.24) | | 7.48(6.00,9.35) |  |  |  |
| Underground front-line | | | | | | | | |
|  | No | <24 | 11(6.47) | | 1 | **4.89**  **(0.72,14.65)** | **0.34**  **(0.06,0.55)** | **1.58**  **(1.07,2.47)** |
|  |  | ≥24 | 54(35.29) | | 7.95(3.94,16.03) |  |  |  |
|  | Yes | <24 | 36(15.58) | | 2.55(1.25,5.20) |  |  |  |
|  |  | ≥24 | 176(50.72) | | 14.39(7.50,27.62) |  |  |  |
| Underground auxiliary | | | | | | | | |
|  | No | <24 | 37(13.75) | 1 | | 0.49  (-2.15.2.84) | 0.06  (-0.26,0.33) | 1.07  (0.78,1.58) |
|  |  | ≥24 | 99(50.25) | 6.73(4.28,10.60) | |  |  |  |
|  | Yes | <24 | 73(22.26) | 1.78(1.47,2.75) | |  |  |  |
|  |  | ≥24 | 382(56.09) | 8.00(5.45,11.73) | |  |  |  |
| Ground worker | | | | | | | | |
|  | No | <24 | 42(16.73) | 1 | | **2.74**  **(0.66,5.36)** | **0.37**  **(0.09,0.57)** | **1.73**  **(1.11,3.00)** |
|  |  | ≥24 | 88(49.44) | 4.47(2.84,7.02) | |  |  |  |
|  | Yes | <24 | 46(20.63) | 1.26(0.78,2.02) | |  |  |  |
|  |  | ≥24 | 285(61.56) | 7.47(5.03,11.08) | |  |  |  |
| Office worker | | | | | | | | |
|  | No | <24 | 24(15.89) | 1 | | 0.89  (-0.83,2.81) | 0.20  (-0.21,0.51) | 1.35  (0.78,3.01) |
|  |  | ≥24 | 66(44.30) | 3.37(1.93,5.91) | |  |  |  |
|  | Yes | <24 | 38(21.23) | 1.17(0.66,2.10) | |  |  |  |
|  |  | ≥24 | 180(54.88) | 4.44(2.65,7.44) | |  |  |  |

RERI, relative excess risk due to interaction;

AP, the attributable proportion due to interaction;

S, synergy index

Bold font is used to highlight statistically significant findings
