# Supplementary material for: Additive interaction of snoring and body mass index on the prevalence of metabolic syndrome among Chinese coal mine employees: a cross-sectional study
Source: BMC Endocr Disord. 2019 Mar 4;19:28. doi: 10.1186/s12902-019-0352-9 (PMC6399959; doi:10.1186/s12902-019-0352-9)
Supplement: Supplementary file 2 — Variable Assignment Table. (DOCX 17 kb) [file 12902_2019_352_MOESM2_ESM.docx]

Variable Assignment Table.

| **Variable** | **Assignment** |
| --- | --- |
| MetS | no = 0, yes = 1 |
| gender | Men=1,women=2 |
| Age group | ≤35 years = 1, 35–45 years = 2, ≥45 years = 3 |
| Monthly Income | ≤4000 (x1 = 0, x2 = 0), 4000–6000 (x1 = 0, x2 = 1), ≥6000 (x1 = 1, x2 = 0) |
| Marital Status | single (x1 = 0, x2 = 0), married (x1 = 0, x2 = 1), divorced (x1 = 1, x2 = 0) |
| Educational Level | bachelor degree or above = 1, junior college and senior high school = 2, junior high school or below = 3 |
| Work Type | heavy physical (x1 = 0, x2 = 0), light physical(x1 = 0, x2 = 1), mental labor (x1 = 1, x2 = 0) |
| Current smoking | no = 0, yes = 1 |
| Alcohol consumption | no = 0, yes = 1 |
| Physical activity level | Inactive (x1 = 0, x2 = 0), minimally active (x1 = 0, x2 = 1), health-enhancing physical activity (x1 = 1, x2 = 0) |
| Workplace | Underground front-line(x1 = 0, x2 = 0, x3= 0), Underground auxiliary(x1 = 1, x2 = 0, x3= 0)，Ground worker(x1 = 0, x2 = 1, x3= 0), Office worker(x1 = 0, x2 = 0, x3= 1) |
| Snoring | Never(x1 = 0, x2 = 0), Occasionally (x1 = 0, x2 = 1), Habitually(x1 = 1, x2 = 0) |
| Body mass index (BMI) | 0（BMI<24）, 1（BMI≥24） |

Monthly income, Marital status, Work type and Physical activity level were analyzed using dummy variable(x1, x2) in multiple logistic regression model. Body mass index (BMI) (kg/m2) was calculated by dividing weight by the square of height.
